# Emerging Image-Guided Navigation Techniques for Cardiovascular Interventions: A Scoping Review

**DOI:** 10.3390/bioengineering12050488

**Published:** 2025-05-02

**Authors:** Majid Roshanfar, Mohammadhossein Salimi, Sun-Joo Jang, Albert J. Sinusas, Jiwon Kim, Bobak Mosadegh

**Affiliations:** 1 Department of Mechanical Engineering, Gina Cody School of Engineering, Concordia University, Montreal, QC H3G 1M8, Canada; m_roshan@encs.concordia.ca; 2 Department of Mechanical Engineering, York University, Toronto, ON M3J 1P3, Canada; mhsalimi@yorku.ca; 3 Section of Cardiovascular Medicine, Department of Medicine, Yale University, New Haven, CT 06519, USA; sun-joo.jang@yale.edu (S.-J.J.); albert.sinusas@yale.edu (A.J.S.); 4 Department of Medicine, Weill Cornell Medicine, New York, NY 10021, USA; jik9027@med.cornell.edu; 5 Dalio Institute of Cardiovascular Imaging, Department of Radiology, Weill Cornell Medicine, New York, NY 10021, USA

**Keywords:** cardiac imaging, image-guided navigation, multimodal imaging, artificial intelligence, interventional cardiology

## Abstract

**Background:** Image-guided navigation has revolutionized precision cardiac interventions, yet current technologies face critical limitations in real-time guidance and procedural accuracy. **Method:** Here, we comprehensively evaluate state-of-the-art imaging modalities, from conventional fluoroscopy to emerging hybrid systems, analyzing their applications across coronary, structural, and electrophysiological interventions. **Results:** We demonstrate that novel approaches combining optical coherence tomography with near-infrared spectroscopy or fluorescence achieve unprecedented plaque characterization and procedural guidance through simultaneous structural and molecular imaging. Our analysis reveals key challenges, including imaging artifacts and resolution constraints, while highlighting recent technological solutions incorporating artificial intelligence and robotics. We show that non-imaging alternatives, such as fiber optic real-shape sensing and electromagnetic tracking, complement traditional techniques by providing real-time navigation without radiation exposure. This paper also discusses the integration of image-guided navigation techniques into augmented reality systems and patient-specific modeling, highlighting initial clinical studies that demonstrate their significant promise in reducing procedural times and improving accuracy. These findings establish a framework for next-generation cardiac interventions, emphasizing the critical role of multimodal imaging platforms enhanced by AI-driven decision support. **Conclusions:** We conclude that continued innovation in hybrid imaging systems, coupled with advances in automation, will be essential for optimizing procedural outcomes and expanding access to complex cardiac interventions.

## 1. Introduction

Advancements in cardiac interventions have revolutionized the treatment of coronary artery disease, structural heart disease, and arrhythmias, significantly improving patient outcomes [1,2]. However, these procedures demand unparalleled precision, particularly in minimally invasive techniques, where direct visualization is limited [3]. Accurate imaging and navigation are indispensable for planning, executing, and evaluating such interventions. Historically, imaging techniques such as fluoroscopy and echocardiography have served as the backbone of cardiac interventions, with advances being made using cardiac computed tomography (CT), magnetic resonance imaging (MRI), and positron emission tomography (PET). However, as the complexity of cardiac diseases grows, there is an urgent need for innovative imaging solutions that provide real-time guidance, decision-making, and patient safety. Emerging image-guided navigation technologies are reshaping this landscape. Integrating multimodal imaging, artificial intelligence (AI), and extended reality (XR) enables unprecedented precision and efficiency in interventional cardiology. These advancements reduce radiation exposure, improve device placement accuracy, and expand treatment options for high-risk patients. Furthermore, they open the door to novel procedures previously deemed unfeasible due to anatomical or technical constraints. This review aims to explore the latest developments in image-guided navigation, highlighting their transformative impact on cardiac interventions. By identifying current challenges and examining future trends, this paper seeks to provide insights into the potential of these technologies to redefine interventional cardiology. While numerous imaging modalities have emerged for cardiovascular interventions, their relative advantages, limitations, and optimal clinical applications vary substantially. This review provides not only a description of these technologies but also a systematic comparison across key parameters, including spatial and temporal resolution, tissue penetration, radiation exposure, and procedural workflow integration. Additionally, we examine how AI is transforming these imaging platforms from passive visualization tools into interactive guidance systems that enhance clinical decision-making and procedural precision. By critically evaluating both technological innovations and implementation challenges, we aim to provide a comprehensive framework for understanding the evolving landscape of image-guided cardiovascular navigation.

### 1.1. Review Methodology

This scoping review was conducted following a structured and reproducible methodology to ensure a comprehensive and balanced evaluation of emerging image-guided navigation techniques for cardiovascular interventions. The reporting of this manuscript adheres to the PRISMA-ScR (Preferred Reporting Items for Systematic Reviews and Meta-Analyses extension for Scoping Reviews) guidelines. Given the rapid advancements in this interdisciplinary field, our approach prioritized the inclusion of recent, high-quality studies spanning clinical cardiology, biomedical engineering, and medical imaging domains.

#### 1.1.1. Search Strategy and Databases

A systematic literature search was conducted across three major databases such as PubMed, Scopus, and IEEE Xplore to ensure broad coverage across biomedical, engineering, and computational research relevant to image-guided cardiac interventions. The search timeframe spanned publications from January 2013 to January 2025, with a deliberate emphasis on recent advances published in the last five years, aligning with the paper’s focus on emerging techniques. We employed a combination of controlled vocabulary (e.g., MeSH terms in PubMed) and keyword strategies tailored to each database. Search terms included but were not limited to the following: *“image-guided cardiac intervention”, “catheter tracking fluoroscopy”, “multimodal cardiac imaging”, “AI in interventional cardiology”, “MRI-guided catheterization”, “OCT-guided PCI”, “intravascular ultrasound guidance”, “real-time navigation cardiovascular”, “robot-assisted cardiac imaging”*, and *“electromagnetic catheter tracking”*. Boolean operators (AND, OR) and truncation techniques were employed to refine the search scope. Reference lists of the included studies were also reviewed to identify any additional relevant records and ensure completeness.

#### 1.1.2. Inclusion and Exclusion Criteria

Articles were selected using predefined inclusion and exclusion criteria. Studies were eligible for inclusion if they satisfied the following:

- Were published in English in peer-reviewed journals or leading international conferences;
- Focused on imaging techniques, navigation systems, or image-guided interventions specifically applied to cardiovascular procedures;
- Demonstrated technical novelty, clinical validation, or translational relevance, such as evaluations of performance, workflow integration, or outcome improvements;
- Included clinical, preclinical, simulation-based, or computational studies with clearly defined methodologies.

Exclusion criteria included the following:

- Editorials, commentaries, abstracts, or opinion pieces without supporting data;
- Studies limited to non-cardiac applications or purely diagnostic modalities without interventional relevance;
- Studies focusing primarily on the development or evaluation of contrast media are excluded, as this review is centered on imaging techniques rather than contrast agent innovation.

#### 1.1.3. Screening and Selection

The initial screening of titles and abstracts was independently carried out by two reviewers to minimize selection bias. Full texts of potentially relevant studies were then retrieved and assessed according to predefined inclusion and exclusion criteria. Any disagreements were resolved through consultation with a third senior reviewer to ensure consistency and objectivity. Through this structured review process, we identified over 180 publications, from which we selected a representative, high-quality, and thematically aligned subset for inclusion.

## 2. Advancements in Imaging Techniques for Cardiac Intervention

The evolution of navigation techniques in cardiac interventions has been fueled by advancements in imaging technologies, computational models, AI, and robotic systems [4,5,6]. These innovations have significantly enhanced procedural precision, safety, and efficacy, enabling clinicians to address complex cardiac conditions more confidently. This section explores the diverse approaches employed in navigation and control during interventional procedures. By reviewing the latest developments and their integration into clinical practice, this section highlights how these techniques are reshaping the landscape of cardiac interventions, opening up the way for more effective and minimally invasive solutions.

### 2.1. Fluoroscopy and X-Ray Imaging Techniques

Fluoroscopy and X-ray imaging are fundamental tools in interventional cardiology, offering the real-time visualization of catheters, guidewires, and devices during minimally invasive procedures. Despite their widespread use, these techniques face notable challenges that limit their effectiveness. High radiation exposure poses risks to both patients and clinicians, particularly during lengthy procedures [7]. Additionally, fluoroscopy provides limited soft tissue contrast, making it difficult to visualize certain anatomical structures without supplemental imaging. The precise tracking of devices such as catheters is another critical challenge, as motion artifacts and overlapping structures in fluoroscopic images can obscure visualization. Emerging strategies are being developed to reduce radiation exposure in fluoroscopy-based procedures. These include AI-driven image enhancement that allows for lower frame rates, fusion imaging with preoperative CT or MRI to minimize real-time fluoroscopy use, and robotic-assisted navigation systems that reduce the need for prolonged manual operation. These approaches aim to make fluoroscopy-guided interventions safer and more efficient. To address these limitations, researchers are developing advanced detection frameworks, navigation systems, and modeling techniques to enhance accuracy, reduce radiation exposure, and improve procedural outcomes.

Ma et al. developed an automatic detection framework to accurately identify catheters in fluoroscopic X-ray images [8]. Leveraging path reconstruction from image tensors derived from a multiscale vessel enhancement filter, their system traces smooth paths along eigendirection vectors to detect catheters and guidewires with high precision. Extensive testing on 7754 X-ray images demonstrated detection errors averaging 0.56 mm for catheters and 0.68 mm for guidewires. Clinical validation further showed its efficacy, with 2D target registration errors of 1.8 ± 0.9 mm for motion compensation and 2.3 ± 0.9 mm for co-registration between 2D X-ray images and 3D MRI models. To overcome navigation challenges in cardiac procedures, researchers developed CathPilot, an innovative cable-driven system that uses 3D cam mechanics to provide clinicians with precise control over medical devices, significantly improving upon traditional methods [9]. To improve catheter localization in endovascular and cardiac procedures, Chang et al. developed a novel system that combines deformable B-spline tube modeling with probabilistic algorithms, enabling the real-time tracking of complex catheter shapes and improving fluoroscopic guidance precision, as validated through phantom and in vivo studies [10]. Recent developments in deep learning have also enabled simultaneous force estimation [11] and catheter segmentation directly from biplane fluoroscopic images. For instance, Fekri et al. proposed Y-Net, a stereo-vision-based architecture that estimates 3D contact forces at the catheter tip using dual-view fluoroscopic images, eliminating the need for embedded sensors [12]. More recently, H-Net extended this concept by introducing an end-to-end multitask framework capable of both stereo segmentation and 3D force regression, demonstrating improved accuracy and computational efficiency [13].

### 2.2. Ultrasound-Based Navigation and Control

**Figure 1:**
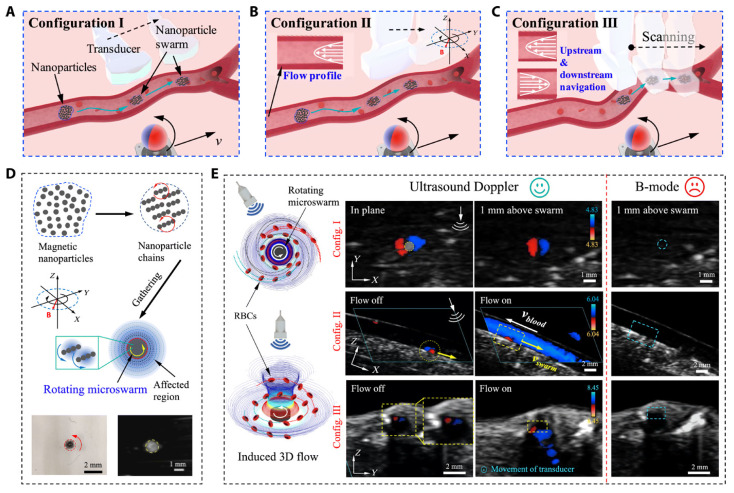
Ultrasound Doppler imaging-guided swarm formation and navigation in blood vessels. (**A**–**C**) Three different configurations for magnetic nanoparticle swarm control and manipulation within vasculature, showing how field orientation affects navigation strategy. (**D**) Formation of nanoparticle chains through magnetic assembly and their targeted delivery capabilities. (**E**) Comprehensive visualization of swarm behavior under different blood flow conditions using B-mode and Doppler imaging for real-time tracking. This multimodal approach enables precise control of therapeutic delivery while providing continuous feedback on swarm positions relative to vessel boundaries, demonstrating superior navigation in complex vascular environments compared to conventional fluoroscopic guidance. Image reproduced with permission from [17].

Ultrasound imaging is a cornerstone of cardiac interventions, providing the real-time visualization of anatomical structures without ionizing radiation. Despite its advantages, several challenges limit its effectiveness in complex procedures. Operator dependency remains a significant issue, as the accuracy and consistency of ultrasound imaging are highly dependent on the clinician’s expertise. Dynamic cardiac environments, such as the motion of heart structures during beating heart surgeries, further complicate imaging by introducing motion artifacts and reducing image clarity. In addition, maintaining stable probe positioning in minimally invasive procedures can be challenging, particularly in transesophageal echocardiogram (TEE) and intracardiac echocardiography (ICE). Despite these challenges, advancements in 3D ultrasound imaging have enhanced anatomical visualization in structural heart interventions, enabling dynamic 3D assessments of heart valve motion, particularly in procedures such as transcatheter edge-to-edge repair (TEER) and transcatheter aortic valve replacement (TAVR). To further enhance ultrasound’s precision and consistency, researchers have developed robotic systems, automated tracking tools, and innovative testing platforms to enhance ultrasound’s precision, consistency, and versatility. Agricola et al. highlighted the pivotal role of TEE in guiding transcatheter structural cardiac interventions, including valve repairs and left atrial appendage closures [14]. The study also emphasized the benefits of integrating echocardiography with other imaging modalities to enhance outcomes and mitigate limitations. However, challenges such as needing specialized imaging expertise and minimizing radiation exposure persist, necessitating further advancements in procedural strategies. Wang et al. proposed a robotic TEE system that automates ultrasound image acquisition along pre-planned paths, guided by electromagnetic tracking and image-based registration [15]. This system significantly reduces operator dependency and improves imaging stability. Phantom experiments validated its performance, achieving a mean positioning error of 10.44 mm in regions of clinical relevance. By integrating robotics with precise navigation, this approach enhances both the reliability and safety of TEE-based procedures. Recent advancements have combined magnetic actuation and ultrasound imaging to enhance guidewire navigation in cardiovascular interventions. Yang et al. developed a magnetically steerable guidewire controlled by mobile electromagnets and guided by ultrasound imaging [16]. Their system uses a computational model to predict guidewire deformation and enables precise, autonomous delivery to target regions, validated in a vascular phantom. This radiation-free alternative to fluoroscopy highlights the potential of ultrasound-based magnetic control to improve precision and safety in minimally invasive procedures. Wang et al. introduced a real-time navigation system for magnetic micro-swarms guided by ultrasound Doppler imaging [17] (see Figure 1). This innovative approach allowed precise swarm tracking and navigation near vessel boundaries, minimizing drag in dynamic blood flow conditions. Validated in porcine coronary arteries ex vivo, the system demonstrates the potential for localized therapeutic delivery in challenging vascular environments, advancing the capabilities of ultrasound-guided interventional techniques.

### 2.3. MRI-Based Navigation and Control

MRI stands as a fundamental tool in diagnostic medicine, combining exceptional soft-tissue contrast with detailed anatomical imaging while eliminating radiation exposure risks. Current research is focused on leveraging the unique features of MRI, such as real-time volumetric imaging and compatibility with advanced robotic systems, to enable precise intra-operative guidance. The ultimate goal is to transform MRI from a diagnostic tool into a fully interactive platform capable of accurately guiding complex interventions. However, challenges such as the high cost of MRI systems, spatial constraints within MRI scanners, and the need for MRI-compatible devices remain barriers to widespread adoption. Several innovative studies have showcased the potential of MRI-based navigation. For instance, Padhan et al. introduced a computational system that integrates real-time image processing with virtual fixtures, dynamically updating visualizations and providing force feedback during procedures [18]. This approach reduced procedural times in simulated transapical aortic valve implantation, illustrating its potential to streamline surgical workflows. Velasco Forte et al. explored the feasibility of MRI-guided catheterization for right and left heart procedures in patients with congenital heart disease. The study demonstrated high procedural success and precise hemodynamic measurements using passive tracking techniques and advanced visualization [19]. Dong et al. tackled real-time catheter tracking under MRI by combining fiber Bragg grating (FBG) sensors with tracking coils [20]. Their system enabled precise shape and positional tracking, demonstrating minimal deviation during autonomous targeting tasks. Similarly, Kensicher et al. explored the use of magnetic field gradients in MRI scanners to control ferromagnetic agents, or MRbots, within blood vessels [21]. This approach represents a novel application of MRI’s capabilities, enabling highly controlled, minimally invasive interventions. Robotic-assisted interventions have also benefited from MRI integration. Miller et al. developed an MRI-compatible robotic arm and valve delivery system for TAVR [22]. The system provided real-time imaging and 3D reconstructions, significantly enhancing accuracy and success rates in preclinical swine models. In dynamic surgical environments, Yeniaras et al. demonstrated the potential of Cine MRI to guide robotic movements during beating heart surgeries, combining preoperative planning with real-time volumetric imaging for enhanced precision [23].

### 2.4. Optical Coherence Tomography (OCT) Technique

**Figure 2:**
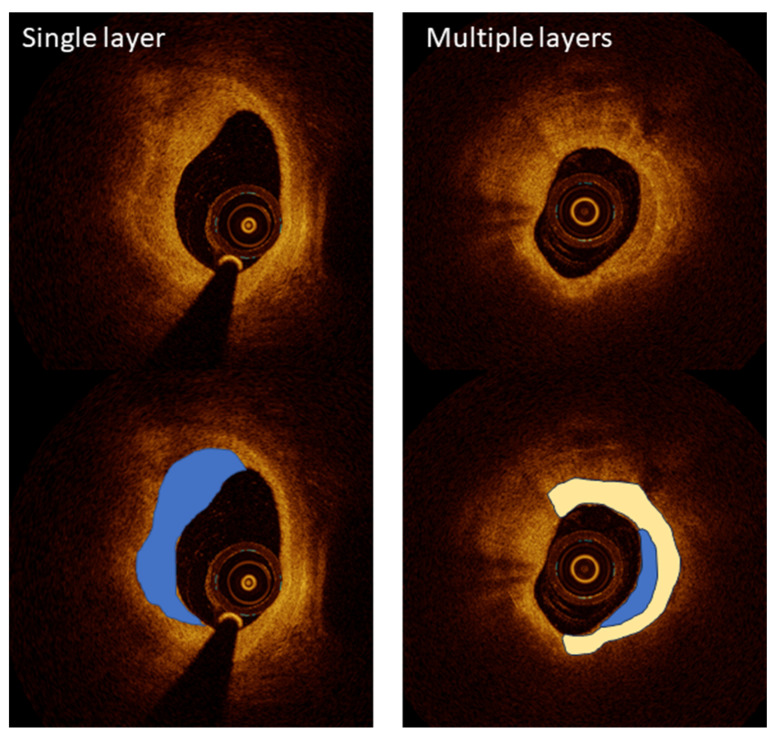
A high-resolution optical coherence tomography (OCT) visualization of atherosclerotic plaque morphology. Upper images show raw OCT cross-sections, while lower images include color-coded interpretations of key plaque features. Left panels demonstrate a single-layered plaque structure (blue overlay) with a crescent-shaped morphology characteristic of healed coronary lesions, providing detailed visualization at 10–20 µm resolution. The right panels illustrate a more complex multi-layered plaque (blue and white overlays), indicating previous rupture and healing cycles that increase vulnerability to future events. This level of microstructural detail significantly exceeds the capabilities of other intravascular imaging modalities, enabling the precise assessment of fibrous cap thickness, a critical determinant of plaque stability. Image reproduced with permission from [26].

OCT has become a critical imaging modality in interventional cardiology, providing ultra-high-resolution images that enable the detailed visualization of coronary artery microstructures. Despite its transformative potential, OCT faces several challenges that must be addressed for broader clinical adoption. One challenge is its limited penetration depth, which restricts its use in imaging larger vessels. Another is the requirement for contrast injection during imaging, which adds complexity and risk, particularly in patients with renal impairment. To overcome these challenges, researchers have leveraged OCT’s unparalleled resolution for specific applications, including plaque characterization, stent optimization, and procedural guidance, ensuring its role as a vital tool in advanced coronary interventions. Kurogi et al. reviewed the clinical applications of OCT-guided percutaneous coronary intervention (PCI), emphasizing its high-resolution imaging capabilities for precise lesion assessment and the optimization of stent implantation [24]. OCT’s ability to characterize lipid-rich plaques and calcium thickness has proven valuable in pre-stent planning, while 3D reconstruction enables the improved management of bifurcation lesions. Although limitations such as high-contrast-volume requirements persist, ongoing advancements offer solutions to expand its clinical utility, underscoring OCT’s transformative impact on PCI outcomes [24]. The LEMON study by Amabile et al. explored the feasibility and safety of using OCT to guide PCI in the left main coronary artery [25]. The results showed that OCT provides superior visualization for optimizing stent apposition and expansion in the mid and distal left main interventions. This pilot study underscores the potential of OCT to enhance outcomes in complex coronary procedures, especially where detailed imaging is critical for procedural success. Yonetsu and Jang demonstrated OCT’s ability to characterize atherosclerotic plaques, differentiating between fibrous caps and lipid cores, a critical factor in assessing the risk of plaque rupture [26] (Figure 2). This high-resolution imaging capability surpasses intravascular ultrasound (IVUS), making OCT indispensable for evaluating vulnerable plaques.

Additionally, OCT’s detailed visualization enables the precise assessment of stent deployment. The technology facilitates evaluating stent apposition and expansion and identifying complications such as tissue prolapse between stent struts, improving procedural outcomes and minimizing risks. A comprehensive analysis of the OCT/IVUS trial conducted by Kang et al. compared the effectiveness of OCT-guided and IVUS-guided PCI in complex coronary lesions [27]. The study, involving 2008 patients, found that both modalities delivered similar outcomes for major endpoints like cardiac death and myocardial infarction. However, OCT-guided PCI significantly reduced major procedural complications (1.7% vs. 3.4%, *p* = 0.03), highlighting its safety benefits in challenging cases. Similarly, Jones et al. analyzed the Pan-London PCI registry, encompassing over 87,000 patients, and found that OCT-guided PCI was associated with improved procedural success and reduced in-hospital major adverse cardiac event (MACE) rates [28]. Long-term mortality was also significantly lower in the OCT-guided group compared to IVUS-guided and angiography-guided groups, underscoring OCT’s contribution to both short- and long-term patient outcomes. In the same way, the iSIGHT randomized trial conducted by Chamie et al. compared OCT, IVUS, and angiography in guiding PCI. The study demonstrated that OCT-guided PCI achieved stent expansion outcomes that were non-inferior to IVUS and significantly better than angiography, with a stent expansion rate of 98.01% [29]. Using an external elastic membrane-based protocol for stent sizing, OCT showed superior precision in optimizing stent deployment, highlighting its potential to enhance procedural outcomes and reduce complications in PCI. To address variability in clinical use, IJsselmuiden et al. undertook a structured evaluation of OCT’s appropriate use [30]. Their findings emphasized OCT’s critical role in specific scenarios, such as assessing stent thrombosis and deploying bioresorbable vascular scaffolds. However, the study also highlighted limitations in its application, suggesting that OCT’s benefits are most pronounced in carefully selected cases, such as addressing unexplained angiographic abnormalities or managing high-risk stent scenarios. Table 1 summarizes the key features of the above imaging modalities, providing a baseline for currently available state-of-the-art cardiac imaging. When comparing conventional imaging techniques, several key performance metrics must be considered in the context of specific interventional requirements. Fluoroscopy offers excellent temporal resolutions (up to 30 frames per second) and a wide field of view, making it ideal for catheter tracking and device deployment, but its limited soft tissue contrast necessitates complementary imaging for detailed anatomical assessment. In contrast, TEE provides superior tissue characterization and the real-time monitoring of functional parameters without radiation exposure, though its invasive nature and operator dependency limit extended use. IVUS bridges some of these gaps, with intravascular views at 100–200 µm resolution that enable precise vessel wall assessment, yet it cannot match the 10–20 µm resolution of OCT for detailed plaque characterization and stent apposition analysis. The clinical decision regarding which imaging modality to employ often depends on the specific procedural phase and anatomical target. For example, while fluoroscopy remains the backbone for catheter navigation through vascular systems, OCT offers superior guidance for coronary stent optimization, and real-time 3D TEE provides crucial monitoring during structural interventions such as TAVR and TEER. This complementary relationship underscores the need for multimodal approaches that leverage the strengths of each technique while mitigating their individual limitations.

To facilitate direct comparisons between imaging modalities, Table 2 provides a systematic evaluation of both conventional and emerging navigation techniques across key parameters, including spatial resolution, temporal resolution, radiation exposure, and demonstrated clinical outcomes, highlighting their relative strengths and limitations for specific cardiovascular interventions.

## 3. Emerging Imaging Techniques for Next-Generation Cardiac Interventions

### 3.1. Near-Infrared Fluorescence (NIRF) and Near-Infrared Spectroscopy (NIRS) in Intravascular Imaging

Near-infrared spectroscopy (NIRS) and near-infrared fluorescence (NIRF) have emerged as powerful tools for intravascular imaging, offering valuable insights into coronary artery disease by enabling the molecular-level detection of lipid-rich plaques, inflammation, and vascular integrity. NIRS has been widely adopted in clinical cardiology, primarily for assessing lipid core burden and guiding PCI. NIRF remains in the preclinical stage, demonstrating the potential for the high-resolution molecular imaging of vascular disease and identifying inflammatory markers within plaques. Chowdhury et al. explored intravascular NIRF imaging as a molecular imaging technique for detecting and characterizing atherosclerotic plaques [31]. By leveraging deeper tissue penetration and reduced autofluorescence, NIRF enhances the detection of molecular markers within plaques, offering greater sensitivity than traditional imaging modalities. The first human intravascular NIRF study validated its potential for clinical coronary imaging, paving the way for targeted applications in diagnosing and managing atherosclerosis. Similarly, Hogue et al. demonstrated the clinical utility of NIRS in monitoring cerebral oxygenation during cardiovascular surgeries [32]. By tracking superficial cerebral cortex oxygenation and integrating oximetry signals with arterial pressure data, NIRS facilitates the real-time monitoring of ischemic thresholds, aiding in personalized patient management and reducing complications in high-risk cardiac procedures. Recent advancements have explored novel NIRF probes and imaging systems to improve visualization and guidance during interventions. Liu et al. introduced a lysosomal viscosity-activatable NIR-II fluorescent probe (NP-V) for intravascular imaging, enabling the real-time detection of oxidative stress and vascular inflammation in ischemic injury models [33]. These findings highlight the potential of NIR-II fluorescence in guiding cardiovascular interventions, particularly for identifying vulnerable plaques and monitoring post-procedural vascular healing.

**Figure 3:**
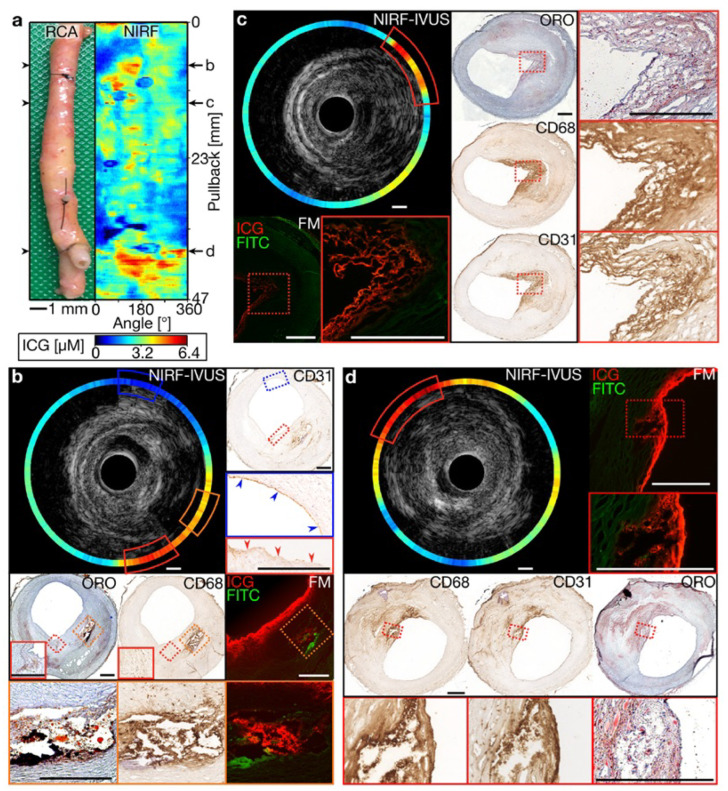
Multimodal molecular and structural characterization of coronary atherosclerosis using integrated NIRF-IVUS imaging. (**a**) Longitudinal view of a human coronary artery with a corresponding NIRF signal map showing the heterogeneous distribution of inflammatory activity (high signal in yellow/red). (**b**–**d**) Cross-sectional NIRF-IVUS images at different arterial locations with corresponding histopathology, demonstrating the precise co-localization of NIRF signals with inflammatory markers (CD68 and CD31) and lipid-rich regions confirmed by histology (ORO staining). This hybrid imaging approach provides a simultaneous assessment of plaque structure (IVUS) and biological activity (NIRF) at a resolution unattainable by either modality alone, enabling more comprehensive risk stratification than conventional angiography or structural imaging. The integration of molecular and anatomical data in a single catheter platform represents a significant advancement for precision-guided coronary interventions. Image reproduced with permission from [34].

As shown in Figure 3, intravascular NIRF imaging combined with IVUS enables the real-time molecular characterization of atherosclerotic plaques, providing key insights into plaque vulnerability and lesion progression [34]. This multimodal approach enhances coronary imaging precision, offering a promising tool for both preclinical research and future clinical applications in interventional cardiology. Beyond coronary imaging, researchers have investigated dual-channel NIRF imaging for surgical applications. Bao et al. developed a dual-channel near-infrared fluorophore system for intraoperative imaging, addressing challenges such as nonspecific uptake and off-target signal detection [35]. Using renally clearable contrast agents, the system provided the precise visualization of tissue boundaries. Although this approach has been predominantly explored in oncological applications, it underscores the potential of dual-channel NIRF for enhanced visualization in complex cardiovascular procedures.

### 3.2. Nuclear Imaging

Nuclear imaging modalities, such as single-photon emission computed tomography (SPECT) and PET, serve as functional imaging techniques to provide crucial insights into myocardial physiology, perfusion, and molecular targets by focusing on physiological and biochemical processes. SPECT is the most widely used clinically in nuclear medicine and is being used to guide targeted drug delivery. SPECT uses gamma rays from the radiotracers accumulated in the specific molecular target. Several radioisotopes such as ^99^mTc, ^123^I, ^67^Ga, ^111^In, or ^201^Tl were used for clinical SPECT imaging. SPECT can be applied to a variety of molecular imaging targets and can also be used for multi-isotope multi-target imaging. Several radionuclides like ^188^Re, ^166^Ho, or ^177^Lu can also be used in image-guided radiotherapy and as probes for monitoring therapeutic efficacy [36]. There has been increasing interest in PET imaging with its higher sensitivity and resolution compared to SPECT. ^18^F-FDG PET/CT offered wide applications for detecting metabolically active diseases in a variety of disease sites and helped in planning radiation therapy [37]. The hybrid imaging between SPECT or PET and CT or MRI improved the detection of the target for biopsy or treatment, with a reduction in registration error and the capability of attenuation correction. Novel developments in SPECT or PET scanners are promising for providing higher spatial and temporal resolutions for the efficient and accurate detection of the target. The most common cardiac molecular targets are transthyretin-related cardiac amyloidosis, which can be detected by ^99^mTc-PYP SPECT, and cardiac sarcoidosis imaged by ^18^F-FDG PET. SPECT and PET can provide the quantitative disease staging and noninvasive monitoring of the disease [38,39]. ^18^F NaF PET is commonly used for detecting atherosclerotic plaques and active valvular calcification, which may help plan coronary and structural heart interventions [22,40,41]. Novel radiotracers targeting myocardial inflammation (matrix metalloproteinase, integrins, and fibroblast activation protein) may help guide drug delivery for myocardial infarction and evaluate remodeling [42].

### 3.3. Multimodalities Imaging Techniques

Multimodality imaging systems have emerged as a transformative approach in interventional cardiology, leveraging the complementary strengths of different imaging modalities to address the complexities of cardiac pathology. The emergence of hybrid imaging systems represents a significant advancement in addressing the inherent limitations of individual modalities. When comparing these integrated approaches, several key advantages become apparent. NIRS-IVUS systems combine the anatomical detail of ultrasound with molecular information on plaque composition, achieving a sensitivity of 89% and specificity of 78% for detecting lipid-rich plaques—significantly outperforming standalone IVUS (sensitivity of 65% and specificity of 64%). Similarly, OCT-NIRF integration enhances the microstructural visualization of OCT (10–20 µm resolution) with the molecular targeting of inflammatory biomarkers, enabling the identification of vulnerable plaques that would remain undetected by conventional angiography or ultrasound alone. The clinical impact of these hybrid systems extends beyond improved diagnostic accuracy to enhanced procedural guidance. In a head-to-head comparison, NIRS-IVUS-guided PCI demonstrated a 47% reduction in major adverse cardiac events at one year compared to angiography-guided interventions, primarily through improved stent sizing and placement. OCT-angiographic co-registration systems have shown similar benefits, reducing geographic miss by 38% and subsequent target lesion revascularization by 42% over conventional approaches. These outcome improvements highlight the transformative potential of multimodal imaging in complex interventional scenarios.

**Figure 4:**
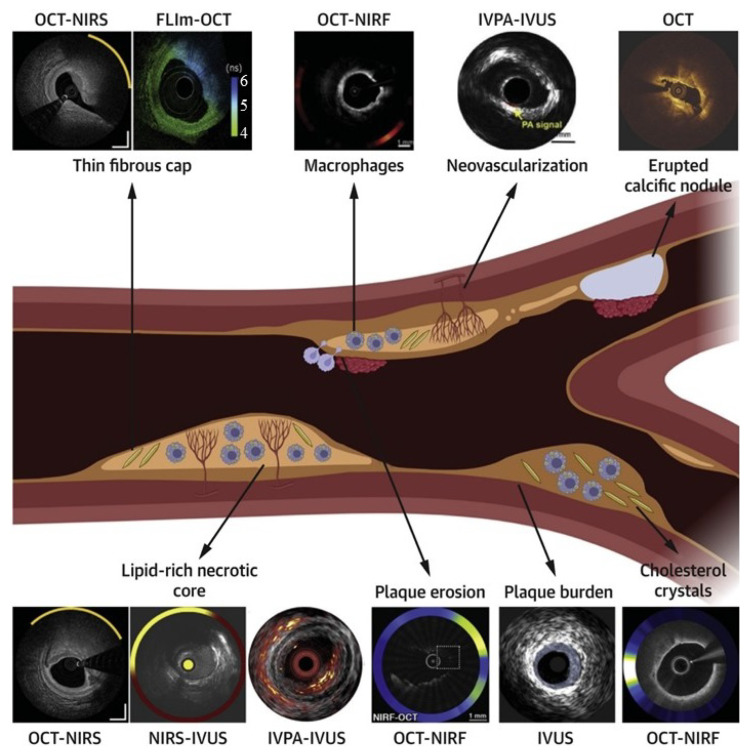
Comprehensive assessment of plaque vulnerability using standalone and hybrid intracoronary imaging modalities. The central schematic illustrates a coronary artery with various plaque features, including a thin fibrous cap, macrophage infiltration, neovascularization, calcification, lipid core, and plaque erosion. Surrounding images demonstrate how specific imaging technologies visualize these features: OCT-NIRS and NIRS-IVUS for lipid detection and cap thickness assessment; OCT-NIRF for inflammatory activity visualization; IVPA-IVUS for neovascularization detection; OCT for calcification identification; and various multimodal approaches for comprehensive plaque characterization. This comparison highlights the complementary nature of different imaging techniques and demonstrates how hybrid approaches provide a more comprehensive assessment than single-modality imaging. Each technology’s unique capabilities address specific diagnostic challenges in vulnerable plaque identification, supporting tailored interventional strategies based on individualized risk assessment. Image reproduced with permission from [43].

Integrating technologies such as IVUS, OCT, and angiography provides a more comprehensive understanding of coronary anatomy and disease. Despite their potential, multimodality systems must overcome challenges such as technological integration and increased procedural complexity. Nevertheless, recent advancements demonstrate their ability to enhance precision, reduce complications, and improve procedural outcomes. Tufaro et al. highlighted the advancements in hybrid intracoronary imaging technologies that integrate IVUS, OCT, and NIRS into multimodality systems [43] (Figure 4). These hybrid catheters provide complementary imaging capabilities, enabling enhanced plaque morphology assessment, high-risk plaque identification, and PCI guidance. Postmortem and clinical studies demonstrated the efficacy of multimodal imaging in improving procedural outcomes and supporting emerging pharmacotherapies targeting atherosclerosis. The NIRVUS trial, conducted by Noori et al., demonstrated the superiority of NIRS and IVUS-guided PCI over angiography-guided PCI in patients with acute myocardial infarction [44]. Imaging-guided PCI achieved significantly higher stent strut coverage (88.7% vs. 72.3%), larger minimal stent areas, and lower incomplete lesion coverage rates. These findings emphasize the importance of combining intravascular imaging modalities to optimize stent deployment and improve procedural outcomes in high-risk AMI cases. A notable example is the OPTICO-Integration II trial by Schneider et al., which investigated the risks associated with post-PCI complications, such as longitudinal geographic mismatch (LGM) and edge dissections [45]. These complications significantly increase the likelihood of adverse events post-PCI. The trial evaluated the efficacy of a novel system combining real-time OCT with angiographic co-registration (ACR). Among 84 patients randomized to ACR-guided PCI, OCT-guided PCI, and angiography-guided PCI, ACR-guided PCI showed a significant reduction in LGM and edge dissections. This superior performance underscores the transformative potential of integrating OCT and angiography for enhanced PCI planning and execution. Lim et al. demonstrated the prognostic value of the lipid core burden index (LCBi), measured using NIRS-IVUS, in predicting slow thrombolysis in myocardial infarction (TIMI) flow after PCI [46]. Their study found that a high LCBi (≥353 for 4 mm segments) was independently associated with reduced TIMI flow and worse clinical outcomes, such as myocardial infarction and stent thrombosis. These findings underscore the utility of NIRS-IVUS in identifying high-risk plaques, optimizing procedural planning, and reducing post-procedural complications.

Hybrid imaging systems that merge IVUS and OCT are another major advancement in multimodality imaging. Sheth et al. reported the first-in-human application of a hybrid imaging catheter capable of simultaneous IVUS and OCT imaging [47]. Featuring a 2.8 F imaging window and a 40 MHz center frequency for IVUS, this catheter was used to treat a 74-year-old male patient recovering from an inferior ST-segment elevation myocardial infarction. Over 90 mm of the right coronary artery was imaged, vividly demonstrating the complementary roles of IVUS and OCT in characterizing atherosclerotic plaque types. This innovation not only provides unparalleled precision in coronary imaging but also paves the way for hybrid systems to become standard tools in coronary interventions. Li et al. further advanced multimodality imaging by developing an ultrafast integrated IVUS-OCT system capable of simultaneous imaging at an unprecedented speed of 72 frames per second [48]. Validation studies using animal models and human cadaveric tissue confirmed the system’s ability to accurately characterize complex atherosclerotic plaques. These findings highlight the potential of hybrid systems to improve procedural guidance and diagnostic precision while reducing the need for multiple imaging setups. Muller and Madder presented a combined OCT and NIRS catheter system for enhanced coronary plaque assessment [49]. This multimodal approach leverages the high-resolution structural imaging of OCT and the lipid detection capabilities of NIRS, providing co-registered data to optimize stenting and identify high-risk plaques. The OCT-NIRS system significantly advances secondary prevention strategies and may pave the way for primary prevention through invasive imaging. Kassab et al. demonstrated the integration of OCT and NIRF imaging using the molecular imaging agent LUM015, which targets cathepsin protease activity (a marker of plaque inflammation) [50]. This multimodal approach leverages the high-resolution structural imaging of OCT and the functional capabilities of NIRF to provide a comprehensive assessment of atherosclerotic plaques. Validated in preclinical and human models, OCT-NIRF imaging highlights the inflammatory microenvironment of plaques, offering a promising tool for identifying high-risk lesions and guiding therapeutic strategies.

### 3.4. Alternative Navigation Methods

While image-guided techniques are predominant in cardiac interventions, several alternative methods provide effective navigation without relying exclusively on imaging. Electromagnetic navigation is one such method, which uses sensors and electromagnetic fields to track the position of catheters and other interventional tools within the heart. The system generates a magnetic field within which sensors detect the precise location and orientation of the catheter [51,52]. This method allows real-time tracking without ionizing radiation, making it a safer option. Another notable method is using catheters with electrode impedance mapping for tracking. This technique involves specialized electrophysiology (EP) catheters equipped with multiple electrodes that measure impedance at various points within the heart. These impedance measurements create detailed anatomical maps that reflect the heart’s structure and function, making it particularly useful for electrophysiological studies and ablation procedures. The real-time tracking and precise localization of the catheter tip provided by impedance mapping avoids the need for traditional imaging and the associated ionizing radiation. However, the quality of impedance data in specific anatomical regions, such as the left atrium or areas with dense scar tissue, can be limited. Magnetic navigation for sensing is another innovative approach that employs magnetic fields to steer and navigate catheters within the heart. Small magnets within the catheter respond to external magnetic fields generated by a navigation system, allowing the precise control of the catheter’s movement. Fiber optic realShape (FORS) technology is another emerging approach for navigation in cardiac interventions [53,54]. FORS technology utilizes fiber optic sensors embedded within catheters to provide real-time shape and position information during procedures. The fiber optic sensors measure curvature, torque, and tip deflection, allowing clinicians to precisely navigate the catheter within the heart. Unlike traditional electromagnetic or magnetic navigation systems, FORS technology does not rely on external magnetic fields or complex imaging systems, reducing the risk of interference and improving procedural efficiency. Additionally, FORS technology offers compatibility with existing catheterization equipment.

## 4. AI-Assisted Image-Guided Navigation

AI redefines imaging navigation in cardiovascular interventions by addressing critical challenges and enhancing procedural precision. By leveraging advanced data analytics, machine learning, and real-time decision-making capabilities, AI significantly improves imaging modalities, facilitates integration with robotics, and supports patient-specific treatment planning. These advancements transform minimally invasive procedures into safer, more efficient, highly personalized interventions. AI-powered imaging systems are making specific, clinically relevant contributions to image-guided navigation, including real-time decision support, image enhancement, and procedural automation. For example, Sibbald et al. demonstrated the utility of AI-enhanced OCT software in improving plaque characterization and guiding stent placement during percutaneous coronary interventions, reducing complications significantly [55]. Jiang et al. and Gandhi et al. highlighted the integration of AI with 3D echocardiography for congenital heart disease interventions, providing clinicians with a clearer visualization of structural defects and enabling personalized care [56,57]. These advancements underscore AI’s potential to refine imaging quality and expand its clinical applications. Kweon et al. developed a deep reinforcement learning framework for automating guidewire navigation in robot-assisted coronary interventions [58]. The framework achieved navigation success rates exceeding 98% in simulated artery phantoms using advanced algorithms and segment-wise learning. The study showcases DRL’s potential to enhance precision, reduce operator dependency, and streamline interventional procedures by integrating human demonstrations and transfer learning. AI also excels in real-time imaging analysis, which is crucial for catheter tracking and device placement during procedures. Ma et al. developed an AI framework for detecting catheters in fluoroscopy, achieving sub-millimeter accuracy and minimizing errors in dynamic surgical environments [59]. Similarly, Unberath et al. showcased how AI enhances 2D/3D image registration, improving guidewire localization and reducing reliance on operator expertise [60]. These studies highlight how AI-driven systems optimize navigation and improve procedural safety.

Traditionally, TEE probe manipulation requires manual operation based on ultrasound image interpretation and cardiac anatomy knowledge. Li et al. introduce a novel approach for guiding TEE probes [61]. They formulate this task as an RL problem, presenting RL-TEE, the first learning-based solution for the 3-DOF control of a TEE probe. Their approach mimics expert echocardiographers’ visual search and navigation strategies, modeling probe-tissue interaction and considering navigation requirements and esophageal compliance. They propose a hybrid deep Q-network model, integrating CNN with self-attention mechanisms to enhance spatial information capture in ultrasound images. Kienzlen et al. explore the automation of catheter navigation in minimally invasive cardiovascular procedures by introducing a concept for automating catheter navigation using RL on simulations [62]. Their results indicate that the agent effectively learns to navigate to predefined target points, facilitating the study of realistic scenarios. Omisore et al. investigate the application of deep RL for motion control in robotic catheterization during percutaneous coronary intervention procedures [63]. Their study proposes a sample-efficient approach with episodic policy transfer, enabling the agent to continuously learn and adaptively tune PID control gains for the axial navigation of endovascular tools. Bian et al. investigate the self-learning of inverse kinematics for the robotic positioning of intracardiac catheters, which is crucial for cardiac ablation procedures [64]. They construct a robotic system and collect a comprehensive dataset using a magnetic tracking system. Employing a genetic algorithm and a feedforward NN, the robot autonomously learns its inverse kinematic model through virtual experiments. The results indicate the successful learning of the kinematic relationship, enabling the accurate prediction of catheter tip positions. The synergy between AI and robotics has led to remarkable progress in autonomous catheter navigation. Liu et al. applied deep reinforcement learning to robotic systems, enabling them to adaptively navigate complex anatomical pathways with high precision [65]. Li et al. proposed an AI-guided robotic ultrasound probe that enhances real-time image acquisition, ensuring accurate visualization and control in minimally invasive procedures [66]. These innovations demonstrate how AI-powered robotics reduce operator dependency and improve procedural consistency.

### 4.1. AI-Enhanced Image Processing and Interpretation

AI is fundamentally transforming image-guided navigation through enhanced processing capabilities that exceed human visual interpretation. Deep learning algorithms now demonstrate remarkable proficiency in automatically segmenting cardiac structures from various imaging modalities, with accuracy approaching expert-level performance. This automated delineation enables the real-time overlay of anatomical boundaries during interventions, helping operators distinguish critical structures even in challenging imaging conditions. Beyond segmentation, AI systems have demonstrated significant advantages in image enhancement and artifact reduction. Convolutional neural networks applied to fluoroscopic images can reduce noise while preserving structural details, effectively decreasing radiation dose requirements by 30–40% without compromising visualization quality. For ultrasound-guided interventions, deep learning algorithms compensate for operator variability by standardizing image acquisition and interpretation, reducing inter-observer variability from 24% to 8% in valvular assessments during structural interventions.

### 4.2. AI-Powered Procedural Guidance and Automation

The integration of AI into procedural workflows extends beyond image processing to active guidance and partial automation. Reinforcement learning algorithms have demonstrated remarkable capabilities in catheter navigation, achieving success rates exceeding 95% in simulated coronary vessels while reducing navigation time by 42% compared to manual control. These systems analyze imaging data in real time to predict optimal catheter paths, accounting for vascular tortuosity and potential obstacles. In electrophysiology procedures, AI-guided mapping systems have shown particular promise, reducing ablation procedure duration by 29% while maintaining equivalent success rates. These systems utilize machine learning to identify arrhythmogenic substrates from integrated electrical and structural data, optimizing ablation target selection. Similar applications in structural interventions employ neural networks to recommend optimal device sizing and positioning based on multimodal imaging inputs, significantly reducing the likelihood of paravalvular leak in TAVR procedures.

### 4.3. Implementation Challenges and Clinical Validation

Despite promising results in research settings, the clinical implementation of AI systems faces substantial challenges. Model generalizability remains a key concern, as algorithms trained on specific patient populations or equipment configurations may perform suboptimally when deployed in diverse clinical environments. Validation studies show a performance degradation of 15–30% when AI systems encounter imaging characteristics that differ significantly from training data. Regulatory pathways for AI integration present additional hurdles, particularly for systems designed for autonomous decision-making rather than decision support. Current regulatory frameworks require extensive validation through multicenter trials, which must demonstrate not only accuracy but also safety and clinical benefit. The dynamic nature of AI algorithms, particularly those designed to learn continuously from new data, poses unique challenges for validation and approval processes designed for static medical devices. Infrastructure requirements for real-time AI implementation, including computational hardware and specialized expertise, represent significant barriers to widespread adoption, especially in resource-limited settings. Successful integration into clinical workflows requires careful consideration of these practical limitations, with graduated implementation approaches that balance technological capabilities with real-world constraints.

## 5. Challenges and Limitations

Despite the remarkable advancements in imaging techniques for cardiac interventions, significant challenges remain that hinder their widespread adoption and full potential. These obstacles span technical limitations, clinical integration, and regulatory hurdles, each requiring targeted solutions to enable the seamless integration of these technologies into routine practice. The operational complexity of advanced imaging systems often leads to increased procedural times, requiring highly precise calibration and setup. Clinically, several challenges affect both patients and healthcare providers. Radiation exposure remains a major concern, particularly with prolonged fluoroscopy use, which can pose long-term risks to patients and clinicians alike. Cost and accessibility further exacerbate these issues, as the high acquisition, maintenance, and operational expenses of advanced imaging systems limit their availability to well-resourced centers. This disparity creates a gap in equitable access to cutting-edge care, particularly in underserved regions. The integration of complex imaging systems into existing clinical workflows also presents difficulties.

These technologies often require significant workflow adjustments, increasing procedural times and sometimes introducing inefficiencies. In addition to technical and clinical challenges, regulatory and training barriers also play a critical role. The regulatory approval process for novel imaging systems, particularly those integrating artificial intelligence, can be slow and cumbersome, delaying their adoption. Meanwhile, the steep learning curve associated with advanced tools, such as robotic-guided ultrasound and hybrid imaging platforms, demands specialized training that many clinicians may lack the time or resources to pursue. Furthermore, the absence of standardized guidelines for the appropriate use of specific imaging modalities can lead to inconsistent application and suboptimal outcomes, highlighting the need for consensus-driven recommendations. Addressing these challenges will require a concerted effort from researchers, clinicians, and policymakers. Technological innovations that simplify system operation, improve image quality, and reduce radiation exposure are critical to overcoming technical barriers. Enhancing clinician training programs and streamlining regulatory pathways will be equally important to ensure that these advancements are both accessible and practical in clinical settings. Finally, fostering collaboration between stakeholders to establish standardized guidelines and equitable access can pave the way for a future where advanced imaging techniques become integral to safe, effective, and minimally invasive cardiac care.

Beyond technical performance, the widespread clinical adoption of advanced image-guided technologies faces several practical barriers. Regulatory approval processes, particularly for AI-driven or autonomous systems, are often lengthy and require extensive multicenter validation to ensure safety and efficacy across diverse patient populations. Cost is another major consideration, as the acquisition, integration, and maintenance of multimodal imaging platforms, robotic systems, and real-time processing tools may limit availability to well-resourced centers. Additionally, these technologies introduce a steep learning curve, requiring targeted physician training and institutional workflow adjustments. Addressing these barriers will be essential to translating innovation into routine clinical practice and ensuring equitable access to advanced cardiac interventions.

## 6. Conclusions and Future Directions

As cardiac imaging continues to evolve, future advancements are set to address current limitations while introducing transformative innovations in interventional cardiology. The next generation of imaging and navigation systems will prioritize enhanced precision, accessibility, and patient-centered care through technological breakthroughs, AI, robotics, wearable devices, and personalized medicine. Together, these developments promise to redefine the standard of care and expand the possibilities of minimally invasive cardiac procedures. Technological innovations are poised to drive the next phase of progress in imaging systems. Non-invasive approaches, such as high-resolution functional MRI and non-contrast-enhanced CT, are emerging as safer alternatives to current methods, reducing procedural risks while maintaining diagnostic accuracy. Simultaneously, augmented reality (AR) and virtual reality (VR) systems are being refined to provide interactive, real-time guidance during interventions [67]. By overlaying detailed anatomical visualizations onto the surgical field, AR/VR systems have the potential to improve precision in device placement and navigation within complex anatomical regions, revolutionizing procedural workflows.

In addition to advances in visualization and guidance technologies, innovations in contrast media are also improving the diagnostic power and safety profile of cardiovascular imaging. For example, carbon dioxide (CO_2_) is being used as an alternative contrast agent in angiography, particularly for patients with renal impairment, and has shown utility in detecting endoleaks during lower limb interventions [68,69]. Similarly, the development of novel agents for ultrasound and MRI enables enhanced imaging of tissue perfusion, inflammation, and fibrosis. These emerging contrast materials extend the capabilities of traditional imaging platforms and will likely contribute to more personalized and effective cardiovascular interventions in the future.

Integrating AI and robotics also represents a pivotal step in advancing the field [70]. Machine learning algorithms are already being developed to provide real-time decision support, helping clinicians assess lesion severity, optimize device selection, and refine procedural strategies based on patient-specific data [71]. Fully automated robotic navigation systems are also on the horizon, promising to reduce operator dependency and enhance procedural accuracy. These robotic systems are designed to autonomously manipulate catheters and devices, minimizing human error while ensuring consistent outcomes, particularly in high-stakes or technically demanding procedures. Wearable technologies and Internet of Things (IoT) devices are emerging as key tools for advancing patient monitoring and remote intervention capabilities [72,73,74,75,76]. Wearables that track cardiac activity and vitals in real time can provide immediate feedback during and after procedures, improving safety and post-operative care. In emergency scenarios, IoT-enabled systems could enable remote navigation, allowing experts to guide interventions from distant locations. This capability has the potential to revolutionize care delivery in underserved or remote regions, bridging gaps in access to specialized expertise and resources.

Looking ahead, the seamless integration of these emerging technologies into clinical workflows will be critical for their success. Innovations such as non-invasive imaging, AI-driven decision support, and wearable technologies must align with the practical needs of clinicians and patients alike. By addressing challenges such as cost, accessibility, and regulatory approval, the field of interventional cardiology can fully embrace these advancements, unlocking new possibilities for precision, safety, and patient-centered care. The future of cardiac imaging is bright, with these trends promising to set new standards for excellence and expand the boundaries of what is possible in minimally invasive interventions, including addressing challenges such as peri-device leaks in cardiac occlusion procedures [77].

## Figures and Tables

**Table 1:** Key features and limitations of conventional image-guided navigation methods in cardiology.

	Modality	Parameters	Explanation
X-ray modalities	Fluoroscopy	Resolution Penetration Depth Depth Estimation Field of View Enhanced Vision Safety and Hazards	Moderate to high resolution depending on the system, ranging from 1- to 2-line pairs per mm. Excellent penetration through soft tissues, limited by bone density. Limited depth perception, as it primarily provides a 2D real-time image. Broad field of view, suitable for dynamic visualization during interventions. Contrast agents enhance visibility, aiding in detailed visualization of vessels and cardiac structures. Exposure to ionizing radiation, requiring careful management to minimize risks.
	Angiography	Resolution Penetration Depth Depth Estimation Field of View Enhanced Vision Safety and Hazards	Similar to fluoroscopy, offering moderate to high resolution. Excellent penetration through soft tissues and vessels. Limited depth perception, primarily providing 2D images. Well suited for visualizing blood vessels and assessing patency. Utilizes contrast agents to enhance visibility and assess vascular structures. Involves exposure to ionizing radiation, and there may be risks associated with contrast agents.
	CT Angiography	Resolution Penetration Depth Depth Estimation Field of View Enhanced Vision Safety and Hazards	High spatial resolution, typically around 0.5 to 1 mm. Limited penetration through bone, excellent visualization of soft tissues. 3D imaging provides detailed depth perception. Comprehensive field of view, capturing detailed anatomy for pre-procedural planning. Iodine-based contrast agents enhance vascular visibility, allowing for detailed assessment of arteries. Involves exposure to ionizing radiation, though advancements aim to minimize radiation dose.
Ultrasound	TTE	Resolution Penetration Depth Depth Estimation Field of View Enhanced Vision Safety and Hazards	Variable, but can achieve high resolution, typically ranging from 1 to 2 mm. Limited penetration through bone, excellent for cardiac imaging. 2D imaging with limited depth perception. Well suited for assessing cardiac structures and functions. Real-time imaging provides dynamic visualization without ionizing radiation. Non-ionizing radiation, considered safe with no known harmful effects.
	TEE	Resolution Penetration Depth Depth Estimation Field of View Enhanced Vision Safety and Hazards	High resolution, often better than transthoracic echocardiography. Excellent penetration due to close proximity of the probe. 3D imaging enhances depth perception. Detailed visualization of cardiac structures and adjacent areas. Provides clearer images, particularly beneficial for guiding interventions near the heart. Generally considered safe, though it involves inserting a probe into the esophagus.
	IVUS	Resolution Penetration Depth Depth Estimation Field of View Enhanced Vision Safety and Hazards	High resolution, typically around 100 to 200 µm. Limited to blood vessels, provides detailed imaging within. 2D cross-sectional imaging, allowing precise assessment of vessel walls. Focused imaging within blood vessels. Direct visualization of vessel walls aids in guiding stent placement. Generally considered safe, though it involves catheterization.
MRI	MRI Imaging	Resolution Penetration Depth Depth Estimation Field of View Enhanced Vision Safety and Hazards	High spatial resolution, typically around 1 to 3 mm. Excellent penetration through tissues, limited by bone. 3D imaging provides detailed depth perception. Comprehensive imaging of cardiac structures. Offers excellent soft tissue contrast without ionizing radiation. Non-ionizing radiation, generally considered safe, but contraindicated in certain conditions.
	MR Angiography	Resolution Penetration Depth Depth Estimation Field of View Enhanced Vision Safety and Hazards	High spatial resolution, typically around 1 to 2 mm. Excellent for vascular imaging. 3D imaging provides detailed depth perception. Excellent for comprehensive vascular assessments. Contrast-enhanced imaging enhances visibility of blood vessels. Non-ionizing radiation, generally considered safe, but contraindicated in certain conditions.
Optical Imaging	OCT	Resolution Penetration Depth Depth Estimation Field of View Enhanced Vision Safety and Hazards	Very high resolution, typically around 10 to 20 µm. Limited to a few millimeters, providing microscopic imaging. 2D cross-sectional imaging with detailed depth perception at a microscopic level. Narrow field of view but offers microscopic details of coronary arteries. Utilizes near-infrared light for exceptional resolution, providing detailed views of vessel walls. Generally considered safe, non-invasive, and does not involve ionizing radiation.
	NIRS	Resolution Penetration Depth Depth Estimation Field of View Enhanced Vision Safety and Hazards	Moderate to high resolution, typically around 1 to 2 mm. Limited to a few millimeters, suitable for assessing arterial plaque composition. Provides information about tissue composition within the imaged depth. Specific to the area of interest, focusing on lipid content within arterial plaques. Near-infrared light to assess lipid content in plaques, aiding in decision-making during interventions. Generally considered safe, non-invasive, and does not involve ionizing radiation.

**Table 2:** Comparison of image-guided navigation techniques for cardiovascular interventions.

Imaging Modality	Spatial Resolution	Temporal Resolution	Radiation Exposure	Real-Time Guidance	Clinical Applications and Outcomes
Fluoroscopy	200–300 μm	Excellent (30 fps)	High (5–15 mSv per procedure)	Excellent	Advantages: Wide field of view, excellent device visibility Limitations: Poor soft tissue contrast, radiation exposure Outcomes: Standard of care for catheter navigation; serves as reference for emerging techniques
CT Angiography	350–500 μm	Limited (75–250 ms)	Moderate to high (3–15 mSv)	Limited, primarily pre-procedural	Advantages: 3D volumetric data, excellent calcification assessment Limitations: Limited intra-procedural use, significant radiation Outcomes: Reduced complications in structural interventions
Transthoracic Echocardiography	0.5–1.5 mm	Excellent (>30 fps)	None	Good	Advantages: No radiation, real-time functional assessment Limitations: Operator dependent, limited windows Outcomes: Improved guidance for structural interventions with a reduction in paravalvular leak
Transesophageal Echocardiography	0.5–1 mm	Excellent (>30 fps)	None	Excellent	Advantages: Superior image quality, real-time 3D capabilities Limitations: Semi-invasive, requires sedation Outcomes: Reduction in procedural complications for structural interventions
Intravascular Ultrasound	70–150 μm	Good (20–30 fps)	None	Good	Advantages: Full vessel cross-section, excellent media- adventitia visualization Limitations: Limited plaque characterization, requires vessel access Outcomes: Reduction in MACE
Optical Coherence Tomography	10–20 μm	Good (15–25 fps)	None	Good	Advantages: Highest resolution, superior stent assessment Limitations: Limited penetration, requires blood clearance Outcomes: Reduction in-stent thrombosis
MRI	1–2 mm	Moderate (10–50 ms frame rate)	None	Limited by acquisition time	Advantages: No radiation, excellent soft tissue contrast Limitations: Limited device visualization, slow acquisition Outcomes: Improved procedural success in congenital interventions
Near-Infrared Spectroscopy	1–2 mm	Goode	None	Moderate	Advantages: Lipid core detection, identifies vulnerable plaques Limitations: No structural information alone, limited to lipid detection Outcomes: Prediction of periprocedural MI
Near-Infrared Fluorescence	0.5–1 mm	Moderate	None	Moderate	Advantages: Molecular imaging, detects inflammatory activity Limitations: Limited clinical validation, requires specific probes Outcomes: Emerging evidence for plaque inflammation assessment
NIRS-IVUS	IVUS: 70–150 μm NIRS: 1–2 mm	Good (20 fps)	None	Good	Advantages: Combined structural and molecular imaging Limitations: Moderate resolution, higher cost than single modality Outcomes: Reduction in MACE compared to angiography-guided PCI
OCT-NIRS	OCT: 10–20 μm NIRS: 1–2 mm	Good (15–20 fps)	None	Good	Advantages: Highest resolution structural imaging with lipid characterization Limitations: Limited penetration, requires blood clearance Outcomes: Reduced edge dissections
OCT-NIRF	OCT: 10–20 μm NIRF: 0.5–1 mm	Good	None	Moderate	Advantages: Combined structural and inflammation assessment Limitations: Limited clinical validation, specialty probes required Outcomes: Emerging evidence for inflammation-directed intervention

Abbreviations: CT—computed tomography; IVUS—intravascular ultrasound; OCT—optical coherence tomography; MRI—magnetic resonance imaging; NIRS—near-infrared spectroscopy; NIRF—near-infrared fluorescence; MACE—major adverse cardiac events; MI—myocardial infarction; fps—frames per second.

## Data Availability

No new data were created in this study.
